# Dyeing and Antibacterial Properties of Chemically Recycled PET Thermal-Bonded Nonwovens Dyed with *Terminalia chebula* Dye

**DOI:** 10.3390/polym12081675

**Published:** 2020-07-27

**Authors:** Joo Hyung Lee, Jong Sun Jung, Seong Hun Kim

**Affiliations:** 1 Department of Organic and Nano Engineering, College of Engineering, Hanyang University, Seoul 04763, Korea; therauss@gmail.com; 2 Research Institute of Industrial Science, Hanyang University, Seoul 04763, Korea; jung.jongsun@gmail.com

**Keywords:** recycled polyester nonwoven, natural dyeing, Terminalia chebula, dyeing condition, antibacterial property

## Abstract

Waste recycling is a necessary step for environmental conservation. To this end, polyester can be easily collected and recycled into end products. To promote the use of recycled polyester, it is important to expand its range of applications. We earlier reported the fabrication of recycled polyester thermal-bonded nonwovens. In this study, recycled nonwoven fabrics were dyed with Terminalia chebula dye without the use of additional mordants. To optimize the dyeing conditions, the dyeing time, dyeing temperature, and liquor concentration were varied, and the color strength, color changes, fastness properties, thermal stability, and morphology were evaluated. Further, the antibacterial activity of the dyed nonwoven was also estimated. T. chebula dyed the colored recycled rapid melting PET fiber (R-RM) nonwoven brown via the dyeing process, and the dyeablity was improved by increasing the dyeing temperature, time, and liquor concentration. The rubbing and sweat fastness properties were found to be excellent. T. chebula dye imparted efficient antibacterial properties to the R-RM nonwovens against *Staphylococcus aureus* and *Klebsiella pneumonia*. The results obtained in this study are expected to broaden the range of natural dyed recycled polyester fabric applications.

## 1. Introduction

There is a growing interest in environmental issues around the world, and significant research on eco-friendly materials is being conducted. Plastics, with numerous advantages such as mass productivity, colorability, and ease of use, and their indiscriminate disposal cause serious environmental pollution problems. For 65 years, from 1950 to 2015, plastic production is estimated at 8300 million tons and plastic wastes is estimated at 6300 million tons. In particular, thpolyester plastics, including polyethylene terephthalate (PET) and polybutylene terephthalate (PBT), have been widely used in textile fibers, nonwovens, films, engineering fields, and packaging materials. Polyester can be easily collected and recycled into end products, unlike other conventional polymer materials [1,2].

In our previous research, we have reported the properties and applications of recycled polyester fiber [3,4,5]. In particular, our group recently conducted a study on the fabrication of thermal-bonded nonwoven fabrics made from chemically recycled bi-component polyester fibers [6]. The bi-component polyester fibers were prepared by co-extruding two different polyester grades in a sheath-core construction; the polyester grades comprise a high melting temperature regular polyester core and a low melting temperature polyethylene terephthalate-polybutadiene terephthalate (PET-PBT) copolymer sheath. Due to the difference in melting temperature between the sheath and core components, the thermal-bonded nonwoven fabrics were prepared without any adhesives and the process was simplified. However, in order to utilize the nonwoven fabrics industrially, further research on dyeing properties is required [7].

In the dyeing industry, synthetic dyes are mainly used due to their advantages, such as variety of colors, ease of dyeing, and excellent fastness. However, synthetic dyes cause a lot of problems due to the accumulation of harmful dye substances in the human body, as well as the water pollution caused due to dyeing wastewater released during dyeing manufacturing processes. In order to ensure environmental and health protection, the production and sales of textile products dyed with 100 kinds of azo dyes are regulated. Therefore, eco-friendly dyeing techniques using natural dyes have attracted the attention of the textile industry worldwide. However, natural dyeing faces problems such as low dyeability, low fastness, and the limitation of color reproduction, as well as environmental pollution caused by the heavy metal mordants used to improve the fastness.

**Figure 1:**
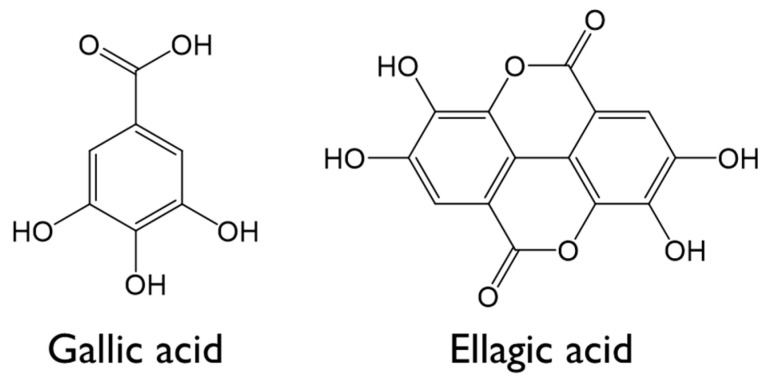
Structures of the main coloring components of *T. chebula* dye.

*Terminalia chebula* (*T. chebula*) is a tropical tall tree native to India; Myanmar; Malaysia; and Sichuan, China. Yellow dye, called *T. chebula* dye or *Myrobalan*, can be extracted from *T. chebula Retzius,* and contains 20%–40% hydrolyzed tannins extracted using methanol. Compounds related to Myrobalan, including gallic acid, ellagic acid, 1,2,3,4,6-penta-O-galloyl-β-D-glucopyranose, chebulagic acid, and chebulinic acid, have been reported; the structures of some of these coloring components are shown in Figure 1. Due to its abundance of tannins, *T. chebula* dye enables the dyeing of fabrics without the use of mordants. In addition, it is also attractive as it is an economical natural dye [8,9,10].

According to the literature [11,12,13,14], natural dyes have mainly been used for dyeing fabrics composed of cellulosic or protein fibers. Many studies have been conducted on the dyeing of natural textiles with various natural dyes, however only a few have been conducted on the dyeing of PET and even fewer on the dyeing of recycled PET with *T. chebula* dye. In this study, *T. chebula* dye was used for dyeing nonwovens composed of recycled PET-recycled PBT bi-component fibers, and the optimal dyeing conditions were investigated. The color strength, color changes, fastness properties, thermal stability, morphology, and antibacterial properties of the dyed nonwoven were also evaluated.

## 2. Experimental

### 2.1. Materials

**Figure 2:**
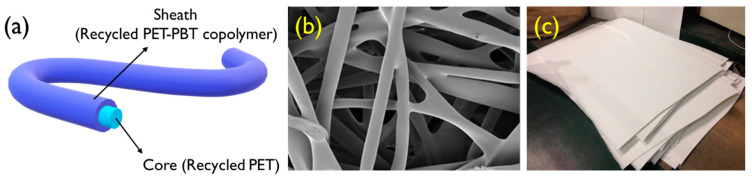
(**a**) Schematic illustration of the recycled bicomponent polyester filament fiber, (**b**) scanning electron microscope image (mag. ×300), and (**c**) photograph of the thermal-bonded nonwoven (R-RM nonwoven) formed therefrom.

*T. chebula* dye originating from India was purchased from the local dye market. Chemically recycled PET-PBT copolymer/PET sheath-core type bicomponent textured fiber, which is called recycled rapid melting PET fiber (R-RM fiber), was used to produce the thermal-bonded nonwovens, as shown in Figure 2. The R-RM fibers were provided by Huvis Co. Ltd., Seoul, Korea, and its specifications are as listed in Table 1. Virgin PET (V-PET) fabric was prepared following the specifications for standard adjacent fabrics (KS K 0905:2015), and 94 tex V-PET yarn was provided by Huvis Co. Ltd., Seoul, Korea.

### 2.2. Preparation of Themal-Bonded Nonwoven

The thermal-bonded nonwovens were prepared as per the procedure reported in our previous research [6]. Briefly, multilayered webs were fabricated by subjecting the R-RM crimped short fibers to carding, and then these were layered cross-directionally. The carded webs were further submitted to a needle punching process with the following conditions: a needle penetration depth of 22 mm, delivery speed of 1.2 m/min, stroke frequency of 700 strokes/min, and barb density of 10,000 needles/m^2^.

The obtained nonwoven fabrics were further heat-treated to produce thermal-bonded nonwovens. The fabrics were prepared at a heat-set temperature of 220 °C and a heating time of 12.5 min; these conditions resulted in optimum mechanical properties in our previous study [6]. The morphology for the prepared R-RM thermal-bonded nonwoven (R-RM nonwoven) is shown in Figure 2b. It was confirmed that the sheath-components of R-RM fibers are well thermally-bonded to each other. As shown in Figure 2c, a large amount of R-RM nonwovens were prepared and used for the dyeing experiments.

### 2.3. Dyeing Procedure

**Figure 3:**
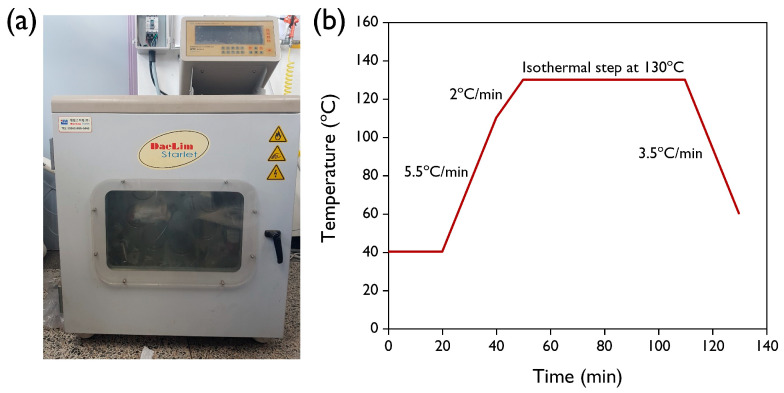
(**a**) The image of the dyeing equipment and (**b**) the temperature profile of the basic dyeing condition.

In order to adjust all the fabric weights to 1 g, R-RM nonwoven and V-PET fabric were cut into 7.5 cm × 7.8 cm and 9.5 cm × 9.5 cm pieces, respectively. The V-PET yarn was cut to a length of 1064 cm. All the dyeing processes were performed in a laboratory infrared (IR) dyeing machine (Daerim Starlet Co., Ltd., Seoul, Korea) using sealed stainless steel dye pots, as shown in Figure 3a. The basic dyeing profile applied is illustrated in Figure 3b. In order to determine the dyeability according to the concentration of dye, the concentration of *T. chebula* was varied to 1, 3, 5, 7, 10, and 13%owf (of the weight of fabric) with a liquor ratio of 1:50 under basic dyeing conditions. The pH of the dye liquor was 3.25. To evaluate the effects of dyeing temperature and time, the temperature of the dyeing bath was gradually increased from 40 °C to the desired temperature (60, 80, 100, and 140 °C), and then that temperature was maintained for the desired dyeing time (20, 40, 60, 80, and 100 min). The dyed nonwovens and fabrics were thoroughly rinsed with deionized water in a liquor ratio of 1:50 for 10 min at room temperature.

### 2.4. Characterization

The fabric color yield was determined by the absorption-to-scattering ratio (*K/S*) calculated by the Kubelka–Munk equation from the average reflectance spectra of the colored fabrics, as measured in the range 400–700 nm by a JS-555 spectrum color meter (Shimadzu UV-240, Shimadzu Corporation, Kyoto, Japan). The absorption-to-scattering ratio is given by Equation (1), as follows:

(1) $$K/S={\left(1-R\right)}^{2}/2R,$$

where *K*, *S*, and *R* are the absorption, scattering, and reflection coefficients, respectively.

L*a*b* (CIE 1976) chromaticities were determined using NF777. 

A light exposure test was carried out according to ISO 105-B02:2014 Textiles—Tests for Color fastness—Part B02: Color fastness to artificial light: Xenon arc fading lamp test. 

The rubbing tests were carried out according to ISO 105-X12:2016Textiles—Tests for Colorfastness—Part X12: Color fastness to rubbing. 

The perspiration test was carried out according to ISO 105-E04:2013Textiles—Tests for Colorfastness—Part E04: Color fastness to perspiration. 

Fourier transform infrared spectroscopy (FT-IR, Nicolet 760 MAGNa-IR spectrometer) were used to determine the structure of the *T. chebula* dye and the dyed textiles over the range of 500–4000 cm^−1^. 

The cross sections of the R-RM nonwovens dyed with *T. chebula* dye at various dyeing temperatures were captured using a digital microscope (VHX-5000, Kenyence Corporation, Tokyo, Japan). Multiple images captured in focus at different heights were composited using the digital fine depth composition function. 

Thermogravimetric analyses (TGA) of the *T. chebula* dye and dyed textile nanocomposites were performed using a Pyris 1 TGA (perkinELmer, Waltham, MA, USA) under an N_2_ gas atmosphere from 30 to 800 °C at 20 °C /min. 

Antibacterial activity tests were carried out according to the KS K 0693-2006 method to detect the bacteriostatic activity on the textile mats. The antibacterial properties of the R-RM nonwovens dyed with *T. chebula* dye were investigated against *Staphylococcus aureus* (Gram-positive bacterium, ATCC 6538) and *Klebsiella pneumonia* (Gram-negative bacterium, ATCC 4532). The cytostatic activity and cytostatic efficiency (%) were calculated as follows:

(2) Cytostatic activity=logB−logA,

(3) $$\mathrm{Cytostatic}\;\mathrm{efficiency}\;\left(\%\right)=\frac{B-A}{B}\times 100,$$

where *A* and *B* represent the average number of bacteria (colony forming unit; CFU) after incubation for 18 h with the bacterial solution derived from the R-RM nonwoven dyed with *T. chebula* dye and a control solution derived from untreated fabric, respectively.

## 3. Result and Discussion

### 3.1. Analysis of the Components of the T. Chebula Dye

**Figure 4:**
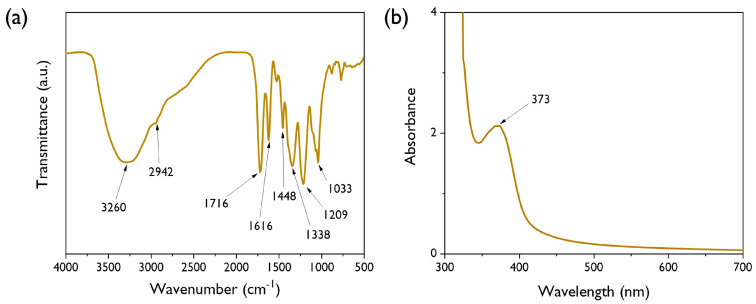
(**a**) FT-IR and (**b**) UV-vis spectra of *T. chebula* natural dye.

The FT-IR spectrum of the *T. chebula* dye used in this study is shown in Figure 4a. The strong band found at 3260 cm^−1^ can be attributed to the phenolic hydroxyl groups (–OH) present in the dye components. The absorption bands found at 1716 and 1616 cm^−1^ indicate the presence of aromatic carbonyl groups. The other bands at 1448, 1033, and 763 cm^−1^ correspond to the stretching vibrations of the –C–C_aromatic_ groups, the aromatic ester –O–, and the distortion vibrations of C=C in benzene rings, respectively. The presence of these characteristic peaks demonstrates that *T. chebula* dye contains a high amount of hydrolysable tannins [15]. These results are supported by a maximum absorbance of 373 nm from a UV-vis analysis, which corresponds to the presence of phenolic and polyphenolic compounds, as shown in Figure 4b. Therefore, it could be speculated that the use of *T. chebula* dye could enable the dyeing of fabrics without the use of additional mordants. 

### 3.2. Effect of Dyeing Conditions on Dyeability

**Figure 5:**
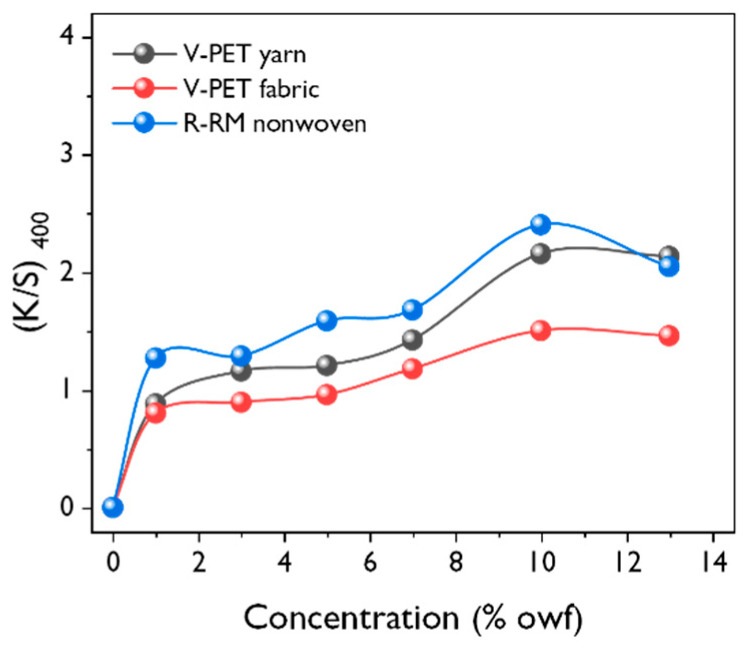
Effect of the dye concentration on the *K/S* value measured at 400 nm (dyeing temperature = 130 °C; dyeing time = 60 min; liquor ratio = 1:50).

The effect of the concentration of dye liquor on the fabrics colored with *T. chebula* dye was investigated. Figure 5 shows the effect of the concentration of dye liquor (1, 3, 5, 7, 10, and 13 %owf) on color strength (*K/S* value) for dyed R-RM nonwovens, V-PET fabrics, and V-PET yarns. The initial dyeing temperature and dyeing time were set to at 130 °C and 60 min, respectively. Based on numerous studies [16,17,18], conventional natural dyes are mainly used for the dyeing of protein fibers because they are easier to dye via both hydrogen and ionic bonds. Unlike other synthetic fibers, PET fibers contain bulky aromatic rings in their primary chains, and hence they exhibit a high hydrophobicity due to the dense fiber structures. Furthermore, PET fibers have high negative surface potentials in aqueous mediums. These properties make it more difficult to dye PET fibers.

The *K/S* values of dyed V-PET fabric and V-PET yarn increased by up to 10 %owf, and the dyeing equilibrium states were reached at subsequent concentrations; in the case of the R-RM nonwoven, the desorption behavior was observed after 10 %owf concentration. Overall dyeability was found in the order of R-RM nonwovens > V-PET yarn > V-PET fabrics. Thus, it is determined that the weaving method affects the dyeability. On comparing the dyeability of V-PET fabrics to that of the V-PET yarn, it is assumed that the *T. chebula* dye cannot easily penetrate into fabrics. Hence, it is also assumed that the R-RM nonwovens have thinner threads, which makes them easier to dye a deep yellow. In conclusion, the *K/S* values increased with increasing concentrations of dye liquors for the three types of samples, and the dyeing equilibrium at 10%owf was found to be the optimal concentration under the basic dyeing conditions.

**Figure 6:**
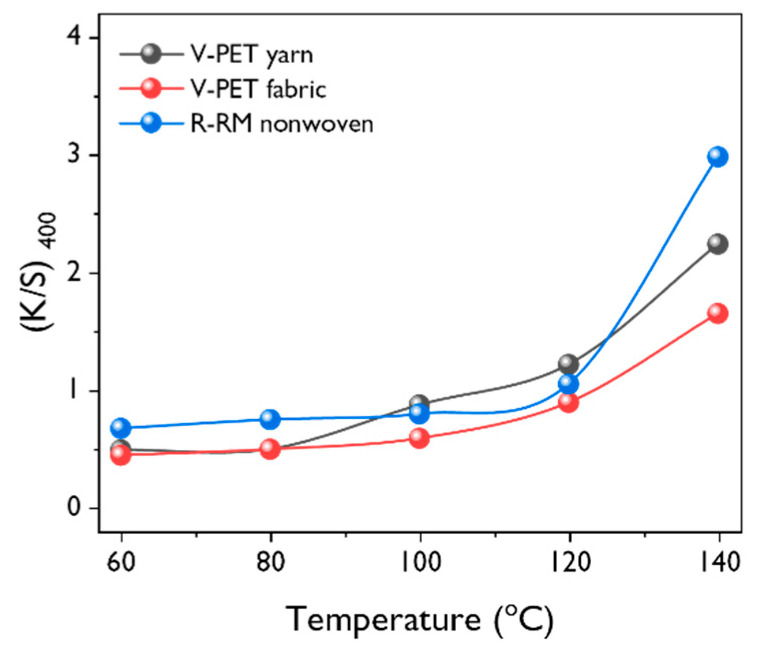
Effect of the dyeing temperature on the *K/S* value measured at 400 nm (dye concentration = 10 %owf; dyeing time = 60 min; liquor ratio = 1:50).

Figure 6 displays the color strength of the dyed R-RM nonwovens, V-PET fabrics, and V-PET yarn under the influence of the dyeing temperature. In the case of hydrophilic fibers, swelling occurs as the temperature of the water increases, which increases the space in the amorphous region of the fibers and allows the dye to diffuse into the fibers. However, hydrophobic fibers, such as polyester, do not swell significantly in aqueous mediums and are usually dyed at approximately 130 °C. In all three dyed samples, the dyeability increased rapidly between 120 and 140 °C. During the dyeing process, water only acts as a medium to transfer dye to the surface of the fabric or yarn. It is considered that the difference in dyeability depends on the thickness and weaving types at 140 °C, and the dyeability also varied in the order of R-RM nonwovens > V-PET yarn > V-PET fabrics. 

**Figure 7:**
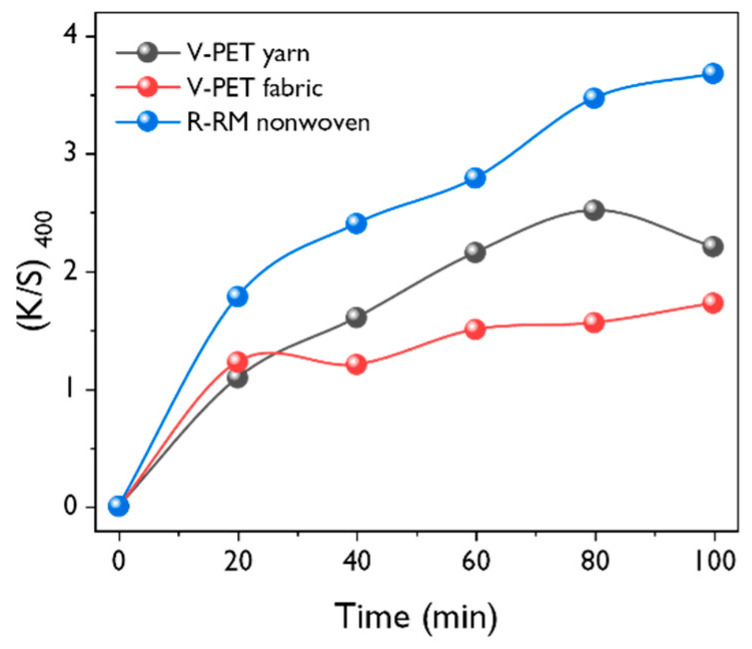
Effect of the dyeing time on the *K/S* value measured at 400 nm (dye concentration = 10 %owf; dyeing temperature = 130 °C; liquor ratio = 1:50).

In order to determine the dyeability according to dyeing times, the dyeing time was varied to 20, 40, 60, 80, and 100 min under basic dyeing conditions, and the *K/S* value is shown in Figure 7. The dyeability with time was shown in the order of R-RM nonwovens > V-PET yarn > V-PET fabrics, which is consistent with those for the optimum concentration of dye liquor and dyeing temperature. The dye in the liquor first diffuses and approaches the surface of the fabric or fiber. Then, it is absorbed at the interface between the surface of the yarn or the fabric and dye liquor. In addition, the adsorbed dye diffuses and spreads inside the fabric, and the dyeing system reaches the dyeing equilibrium. In the case of R-RM nonwovens, the rate of diffusion of the dye adsorbed at the interface between the surface of the nonwoven and dye liquor is faster than that of fabrics and yarn. The dye ability increases rapidly until the dyeing time of 80 min, and the increase continued after that dyeing time. Hence, the dyeing time of 100 min is considered as an optimum condition. In the case of V-PET yarn, 60 min was enough for obtaining the highest K/S value. When the dyeing is continued, the K/S value is observed to slightly diminish. 

**Figure 8:**
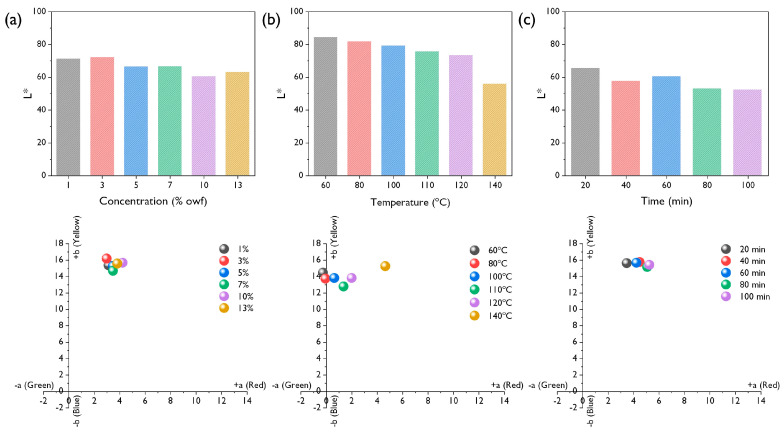
Colorimetric data of the R-RM nonwoven dyed with *T. chebula* dye in relation to (**a**) the dye concentration, (**b**) temperature, and (**c**) dyeing time.

The corresponding L* (brightness), a* (red-green), and b* (yellow-blue) values and apparent color of the R-RM nonwovens, V-PET fabrics, and V-PET yarn dyed with *T. chebula* dye are shown in Figure 8 and Table 1, respectively. In Figure 8a, at a dye concentration of 10 %owf the L* value was the lowest, indicating the best dyeability. There was no significant change in the a* and b* values with respect to the dye concentration. In Figure 8b, the red-green hue became stronger as the dyeing temperature increased, and at the dyeing temperature of 140 °C it can be seen that the brightness was drastically lowered. Therefore, in the R-RM nonwovens it can be seen that dyeability is most affected by temperature change [19]. In Figure 8c, L* showed the lowest values, with little difference between the dyeing times of 80 and 100 min, and no significant changes in the a* and b* values were observed. These results can be more clearly observed in the apparent color comparison, as shown in Table 2. Compared to the untreated R-RM nonwovens, which are almost white, deeper shades were obviously observed upon increasing the dye concentration, dyeing temperature, and dyeing time. These trends are also consistent with the L*, a*, and b* values mentioned earlier. In particular, the most effective dyeability was shown at 10 %owf, 140 °C, and 80 min, which supports the results of the *K/S* color strength analysis. Based on the dyeability analysis results, it was confirmed that the effective dyeing of R-RM nonwovens using *T. chebula* dye could be achieved by dyeing condition control.

### 3.3. Color Fastness

In general, the color of textiles gradually fades on exposure to sunlight, sweat, washing, and rubbing. In particular, most natural dyes show a lower color fastness than synthetic dyes. Table 3 presents the light, rubbing, and sweat color fastness properties of dyed R-RM nonwovens at the optimum dyeing condition (dye concentration = 10 %owf; dyeing temperature 140 °C; dyeing time 80 min). The light color fastness of the optimum sample was shown to be of grade 2–3. Based on many previous studies [20,21,22], it is reported that the light color fastness for fabrics dyed with natural dyes without any mordant shows a poor grade; however, this study showed a slightly higher light color fastness without the use of mordants. The tannin contained in *T. chebula* dye may affect the light color fastness of the optimum sample, as it is known that mordant treatment with tannins can improve the light fastness in naturally dyed fabrics. The result of the rubbing fastness showed the highest grade of 4-5. Due to the high crystalline structure of R-RM nonwovens, the sweat fastness is considered to be excellent at grade 4-5 in both acid and alkaline environments. 

### 3.4. Morphology

**Figure 9:**
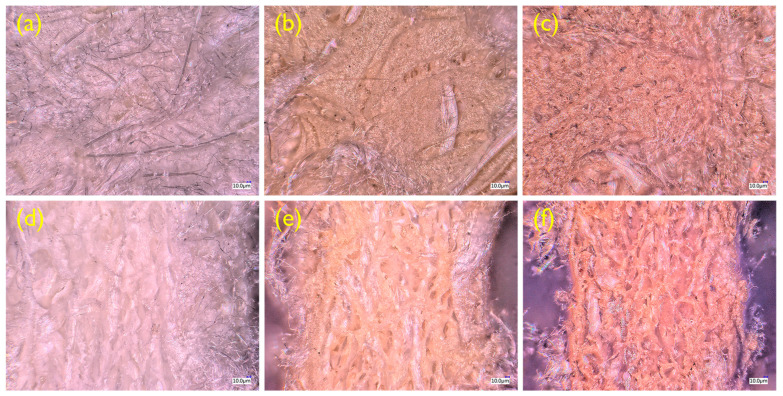
Focus stacked images of the surface and cross-section for (**a**,**d**) R-RM nonwoven, (**b**,**e**) R-RM nonwoven dyed at 100 °C, and (**c**,**f**) R-RM nonwoven dyed at 140 °C.

In order to investigate the internal dyeing state of the dyed thermal-bonded nonwovens according to temperature, the cross-sections of the samples were photographed with a digital optical microscope. Since general optical microscopes have a shallow depth of field, it is difficult to accurately capture the cross section of the nonwoven in a single photograph. Therefore, dozens of photos taken at 3 μm intervals were manipulated into a single image using the focus stacking function, as shown in Figure 9. It is confirmed that the R-RM nonwovens were uniformly colored inside as well as on the surface with the temperature increase due to the diffusion of the *T. chebula* dye. However, a slight thermal shrinkage occurs when they are dyed at 100 and 140 °C (Figure 9d–f).

### 3.5. TGA Analysis

**Figure 10:**
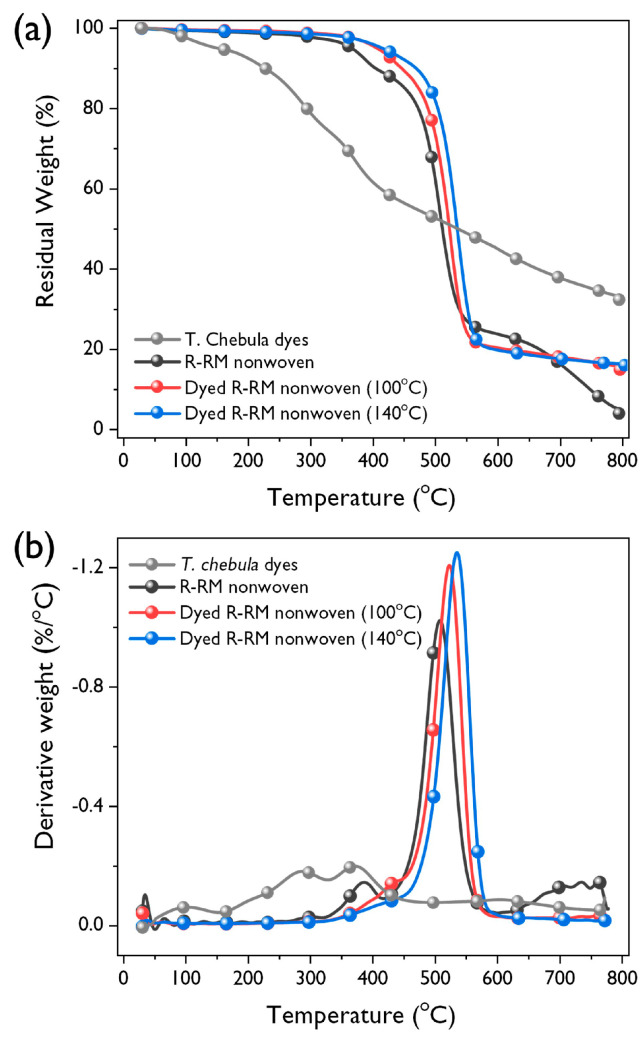
(**a**) TGA thermograms of and (**b**) derivative curves of *T. chebula* dye, R-RM nonwoven, R-RM nonwoven dyed at 100 °C, and R-RM nonwoven dyed at 140 °C.

Figure 10 illustrates the comparison of thermal stability for *T. chebula* dye, R-RM nonwovens, and R-RM nonwovens dyed with *T. chebula* dye at 100 and 140 °C. In the case of the *T. chebula* dye, an approximately 5% weight loss was observed below 150 °C, under a nitrogen atmosphere. This is due to the release of acetic acid and water from the tannic acid in the dye. The dye exhibited a high residual weight of 32% at 800 °C and the char showed intumescence, which is characteristic of tannic acid [23]. The R-RM nonwoven (Table 4) has a thermal decomposition temperature of 367 °C at 5% weight loss (T_5%_) and a peak thermal decomposition temperature (T_D_) of 508 °C. On dyeing, there was slight increase in the T_5%_ and T_D_. At 140 °C, the T_5%_ and T_D_ were 415 and 536 °C, respectively. In fact, tannic acid can be considered to be moderately flame-resistant due to its high total heat release and heat release capacity. Several studies have been reported to improve the thermal stability of polymer through blends with tannic acid [24,25]. It is interesting to note that the dyeing using only natural dyes containing tannic acid slightly enhances the thermal stability of the recycled polyester thermal-bonded nonwovens. These results may be attributed to the thermal barrier effect of *T. chebula* dye, which is well attached to the surface of the R-RM nonwoven. This is further supported by the higher thermal stability of R-RM nonwovens dyed at 140 °C than that of the R-RM nonwovens dyed at 100 °C.

### 3.6. Antibacterial Activity

**Figure 11:**
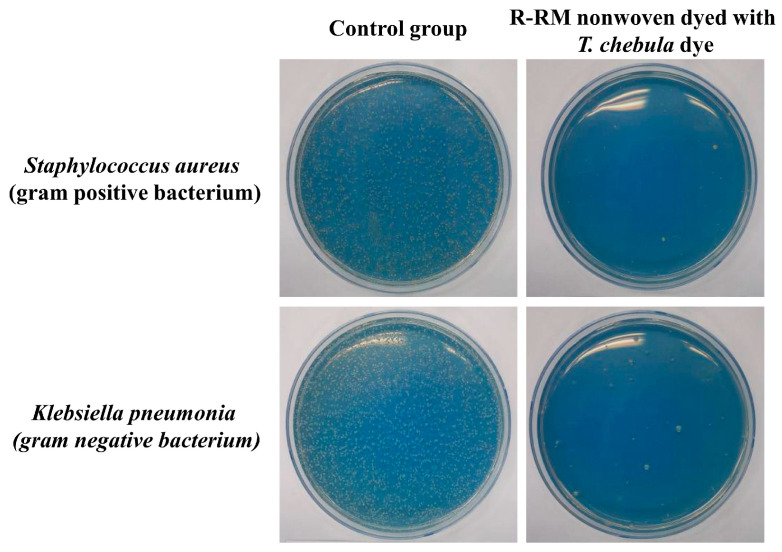
Images of the antibacterial test of the R-RM nonwoven dyed with *T. chebula* dye.

Antibacterial tests for the R-RM nonwoven dyed with the *T. chebula* dye and the control group were conducted using representative Gram-positive (*S. aureus*) and -negative (*K. pneumonia*) bacteria [26]. Table 5 summarizes the cytostatic activity and efficiency of the R-RM nonwoven dyed with *T. chebula* dye according to bacteria kinds, and the images are shown in Figure 11. In the case of the untreated R-RM nonwoven, no bacterial reduction rate was observed for *S. aureus* and *K. Pneumonia* bacteria. The calculated cytostatic efficiency of the R-RM nonwoven dyed with *T. chebula* dye is 99.9 against both *S. aureus* and *K. pnuemoniae*. This can be attributed to the antibacterial properties of the tannin ingredients in the *T. chebula* dye [27].

## 4. Conclusions

In this research, recycled rapid melting nonwovens were dyed using *T. chebula* dye without the use of additional mordants to prepare the colored and functionalized nonwovens. For a comparison of dyeability, virgin PET yarn and virgin PET fabric dyed with *T. chebula* dye were also prepared under the same conditions. For each sample, the dyeability, colorfastness, thermal stability, morphology, and antibacterial properties were evaluated. The dye was used to color R-RM nonwovens brown via the dyeing process, and the dyeablity was improved by increasing the dyeing temperature, time, and liquor concentration. The rubbing and sweat fastness properties were found to be excellent even though no mordant was used. As a result of morphology analysis, it was confirmed that the R-RM nonwoven was uniformly colored inside as well as on its surface at a high dyeing temperature. The R-RM nonwoven showed a high thermal stability due to the thermal barrier effect of the dye. The dye imparted efficient antibacterial properties to the R-RM nonwoven, particularly against *S. aureus* and *K. pneumonia*. Overall, it is considered that R-RM nonwovens dyed with *T. chebula* dye may have a potentially wide range of recycled material applications, such as in automotive interiors, where antibacterial properties are required.

## Figures and Tables

**Table 1:** Specification of the recycled rapid melting PET fiber (R-RM) nonwoven.

Characteristics	R-RM Fiber
Linear density (denier)	4.7
Tenacity (cN/tex)	32.5
Elongation (%)	52.3
Modulus at 1% (cN/tex)	172.2
Crimp number (ea/2.5 cm)	12.0
Crimp stability (%)	57.3

**Table 2:** Images of the dyed samples according to various dyeing conditions.

**Untreated**	**R-RM** **Nonwoven**					
	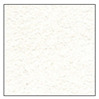					
**Dye conc.** **(%owf)**	**1**	**3**	**5**	**7**	**10**	**13**
	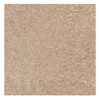	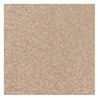	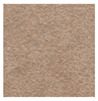	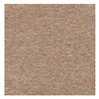	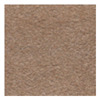	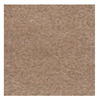
**Temperature** **(°C)**	**60**	**80**	**100**	**120**	**140**	
	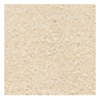	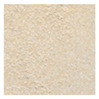	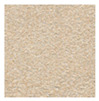	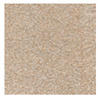	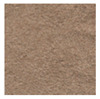	
**Time** **(min)**	**20**	**40**	**60**	**80**		
	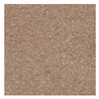	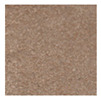	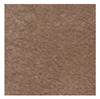	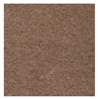		

**Table 3:** Results of light, rubbing, and perspiration fastness for the dyed R-RM nonwoven.

Colorfastness				R-RM Nonwoven
**Light**	**Color change**			2–3
**Rubbing**	**Dry**			4–5
	**Wet**			4
**Perspiration**	**Acidic**	**Color change**		4–5
		**Stain**	**Cotton**	4–5
			**PET**	4–5
	**Alkalic**	**Color change**		4–5
		**Stain**	**Cotton**	4–5
			**PET**	4–5

**Table 4:** Thermal degradation parameters of the *T. chebula* dye and dyed R-RM nonwoven.

	TGA in N_2_ Atmosphere		
	T_5%_ (°C) *^a^*	T_D_ (°C) *^b^*	Char Yield (%)
*T. chebula* dye	150	370	32
R-RM nonwoven	367	508	4
R-RM nonwoven dyed at 100 °C	407	524	14
R-RM nonwoven dyed at 140 °C	415	536	16

*^a^* Initial thermal degradation temperatures at 5% weight loss; *^b^* Temperatures at maximum degradation rates.

**Table 5:** Antibacterial ability of the R-RM nonwoven dyed with *T. chebula* dye according to bacteria strains.

Sample	R-RM Nonwoven	
Bacteria Type	*S. aureus* (ATCC 6538)	*K. pneumonia* (ATCC 4352)
Initial average number of bacteria (CFU ^1^)	1.8 × 10^4^	1.8 × 10^4^
A ^2^	1.8 × 10^4^	1.8 × 10^4^
B ^3^	9.2 × 10^6^	3.8 × 10^7^
Cytostatic activity	2.7	3.3
Cytostatic efficiency (%)	99.9	99.9

^1^ CFU: colony-forming unit; ^2^ A: average number of bacteria after the antibacterial test for the dyed R-RM nonwoven; ^3^ B: average number of bacteria after bacterial culture for the untreated fabric.
